# Fruit-80: A Secure Ultra-Lightweight Stream Cipher for Constrained Environments

**DOI:** 10.3390/e20030180

**Published:** 2018-03-08

**Authors:** Vahid Amin Ghafari, Honggang Hu

**Affiliations:** Key Laboratory of Electromagnetic Space Information, Chinese Academy of Sciences, School of Information Science and Technology, University of Science and Technology of China, Hefei 230026, China

**Keywords:** stream cipher, ultra-lightweight, lightweight, Grain, small-state, NFSR, LFSR, hardware implementation

## Abstract

In Fast Software Encryption (FSE) 2015, while presenting a new idea (i.e., the design of stream ciphers with the small internal state by using a secret key, not only in the initialization but also in the keystream generation), Sprout was proposed. Sprout was insecure and an improved version of Sprout was presented in FSE 2017. We introduced Fruit stream cipher informally in 2016 on the web page of IACR (eprint) and few cryptanalysis were published on it. Fortunately, the main structure of Fruit was resistant. Now, Fruit-80 is presented as a final version which is easier to implement and is secure. The size of LFSR and NFSR in Fruit-80 is only 80 bits (for 80-bit security level), while for resistance to the classical time-memory-data tradeoff (TMDTO) attacks, the internal state size should be at least twice that of the security level. To satisfy this rule and to design a concrete cipher, we used some new design ideas. It seems that the bottleneck of designing an ultra-lightweight stream cipher is TMDTO distinguishing attacks. A countermeasure was suggested, and another countermeasure is proposed here. Fruit-80 is better than other small-state stream ciphers in terms of the initialization speed and area size in hardware. It is possible to redesign many of the stream ciphers and achieve significantly smaller area size by using the new idea.

## 1. Introduction

Nowadays the need for secure lightweight symmetric ciphers is obviously more than that of eSTREAM project time. In the Internet of things (IoT) many constrained devices (e.g., wireless sensor network (WSN), Radio-frequency identification (RFID), and Wireless Body Area Network (WBAN)) should be securely connected to the internet.

Three stream ciphers (Trivium [1], MICKEY 2.0 [2], and Grain-v1 [3]) have been introduced in the hardware profile of the eSTREAM project. Grain-v1 uses both a linear feedback shift register (LFSR) and a non-linear feedback shift register (NFSR). The linear section guarantees good statistical properties and a large period, while the nonlinear section protects against the attacks that can be mounted against a linear cryptosystem. A related-key attack based on the weakness in the initialization procedure of Grain-v1 was proposed in [4]. Grain-128 was introduced in 2006 [5], and some attacks were suggested to it [4,6,7,8,9,10,11]. Indeed, Grain-128 is not secure as expected. Grain-128a was proposed in 2011 [12]. Although some attacks have been applied to Grain-128a [13,14], it is still good from the practical point of view.

In FSE 2015 a new idea, the design of stream ciphers with the shorter internal state, was introduced [15]. Sprout stream cipher was proposed as an instance based on the new idea. A short while after Sprout was introduced, many attacks were published against it [16,17,18,19,20,21]. Although it has been found that Sprout is insecure, it has a new idea to design stream cipher with smaller area sizes. Its new idea is to use key not only in the initialization procedure but also in the keystream generation. We introduced Fruit stream cipher informally in 2016 on the web page of IACR (eprint) and few cryptanalysis were published on it [22,23,24,25]. Fortunately, the main structure of Fruit was resistant to key recovery attacks, and the published key recovery attacks were related to the unsuitable choice of some parameters in the design. An improved version of Sprout, Plantlet, was introduced in FSE 2017 [26]. Now, Fruit-80 is presented as the final version that is easier to implement and secure.

It seems that the bottleneck of designing a secure ultra-lightweight stream cipher is TMDTO distinguishing attacks. TMDTO distinguishing attacks were successfully applied to all of the small-state stream ciphers (i.e., Sprout, Fruit, and Plantlet [24]). Designers of Plantlet ruled out the TMDTO distinguishing attack against their cipher. They stated that the attack needs so many resources, and it can only distinguish the keystream of ciphers from random sequences [26]. The publishers of TMDTO distinguishing attacks stated:

*“We would like to point out that, in our opinion, the existence of distinguishing attacks with a complexity below that of exhaustive key search should not be a knock-out criterion for stream ciphers targeting ultra-lightweight applications because such attacks might actually be tolerable depending on the application scenario”*.

[24]

Nevertheless, as the attack complexity is lower than that of brute force attack, two countermeasures in the design of Fruit-80 are proposed. It is possible based on the conditions to employ one of them. The first one, which is permanently IV mixing, was proposed in [24]. We propose the second countermeasure that is producing limited numbers of keystream bits under every key.

The storing of key bits for reuse by different IVs is essential for most applications, and it is also necessary to store a key in a fixed memory in some applications (in these cases, one fixed key is sufficient forever, e.g., in RFID systems or SIM cards for mobile phones). It is a valuable idea that the stored key is also used in the design as a part of the internal state. This idea helps designers to extend the internal state to the key bits. Thus, it is possible to design ciphers with significantly smaller area sizes, or, the idea helps us to achieve a bigger internal state and stronger ciphers in the same area size.

The necessary condition for a stream cipher to be resistant to TMDTO attacks is that the internal state size should be at least twice that of its security level, such as Trivium, MICKEY 2.0, and Grain-v1. We show how we can exploit key in a design to achieve shorter internal state. We think that it is a new generation in the design of stream ciphers. As we show, Fruit-80 is secure and ultra-lightweight and can be more resistant than Grain-v1 to some attacks such as Cube attacks and related-key attacks. Unlike the Grain family and Sprout, there is no weak key-IV in Fruit-80.

Fruit-80 is better than Plantlet, Sprout, and Lizard ciphers in terms of the initialization speed. Fruit-80 (similar Grain-v1) needs 160 clocks for initialization, while Plantlet, Sprout, and Lizard need 320, 320, and 256 clocks for initialization, respectively. In addition, Fruit-80 is better than Fruit from the applicability point of view. Fruit-80 requires access to three key bits sequentially, while the round key function of Fruit uses six key bits randomly. In the round key function of Fruit-80, an 80-bit key is divided to three independent parts, and it needs to access one bit of each part cyclically in every clock. Thus, it is feasible to implement the round key function of Fruit-80 in internal memories (i.e., integrated into ASIC) and external memories efficiently.

Because Grain-v1 is lightweight and there is no practical attack to it, we introduce Grain-v1 as a basis for identifying the ultra-lightweight stream ciphers. We call a stream cipher with less than 80% GE (gate equivalents) of the hardware implementation of Grain-v1 (in the same condition such as same technology process and the same compiler) as an ultra-lightweight stream cipher. A stream cipher with less than 1K GE has been called ultra-lightweight cipher in [17]. As GE is dependent on many factors, (and different implementations of a cipher have the different GEs), our definition is more accurate.

We present the hardware implementation results and compare the area sizes of some stream ciphers. Fruit-80, Plantlet, Lizard, and Grain-v1 require 960, 996, 1218, and 1270 GE for implementations in TSMC 0.18 μm technology, respectively. These results show that Fruit-80 is the lightest small-state stream cipher and the area size of Grain-v1 is about 32% bigger than that of Fruit-80. These results are expected because the size of the internal states of Fruit-80, Plantlet, Lizard, and Grain-v1 are 87, 101, 121, and 160 bits, respectively. We call a stream cipher with internal state size less than twice of the security level as a small-state stream cipher. Thus, Lizard is a small-state stream cipher because its internal state is 121 bits for 80-bit security level [27]. Note that Lizard is not an ultra-lightweight stream cipher according the definitions.

The rest of the paper is structured as follows. The design of Fruit-80 and the design criteria are presented. Then, we show that Fruit-80 is resistant to known attacks. Finally, we discuss the hardware implementation of Fruit-80.

## 2. The Design of Fruit-80

**Figure 1:**
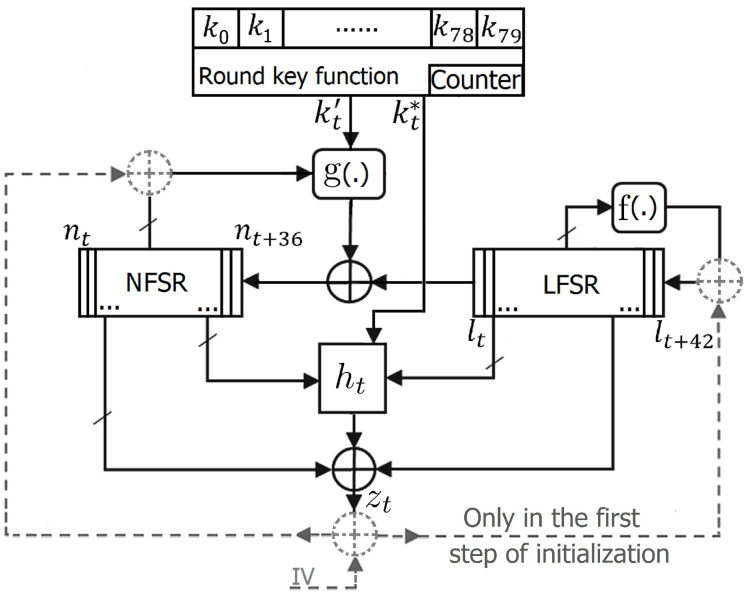
The Block Diagram of Fruit-80.

The internal state consists of a 43-bit LFSR $\left(l_{t},\ldots ,l_{\left(t+42\right)}\right)$, a 37-bit NFSR $\left(n_{t},\ldots ,n_{\left(t+36\right)}\right)$, and a 7-bit counter ($Cr:(c_{t}^{0},\ldots ,c_{t}^{6})$). A general view of Fruit-80 is presented in Figure 1. Inputs of Fruit-80 are a 80-bit secret key (*K*: $\left(k_{i},0\leq i\leq 79\right)$) and a 70-bit public Initial Value, (IV: $\left(v_{i},0\leq i\leq 69\right)$). The maximum number of the keystream bits that can be produced from one key and IV is $2^{43}$ bits. It is not acceptable to reuse IVs (i.e., the use an identical IV with different keys is prohibited).

Now we explain each part of the cipher in details:

### 2.1. Round Key Function

We define some indices of the key for using in the round key function. The indices are dependent on Cr counter and they change in each clock. We introduce $r=(c_{t}^{0}c_{t}^{1}c_{t}^{2}c_{t}^{3})$, $p=(c_{t}^{1}c_{t}^{2}c_{t}^{3}c_{t}^{4}c_{t}^{5})$, and $q=(c_{t}^{2}c_{t}^{3}c_{t}^{4}c_{t}^{5}c_{t}^{6})$. We combine 3 bits of the key to obtain the bits of the round key for **g** function ($k_{t}^{\prime }$) and round key for h function ($k_{t}^{*}$) in each clock as follows.

(1) $$\begin{array}{cc} k_{t}^{\prime }= & k_{r}\cdot k_{\left(p+16\right)}\cdot k_{\left(q+48\right)}⊕k_{r}\cdot k_{\left(p+16\right)}⊕k_{\left(p+16\right)}\cdot k_{\left(q+48\right)}\\ & ⊕k_{r}\cdot k_{\left(q+48\right)}⊕k_{\left(p+16\right)} \end{array}$$

(2) $$\begin{array}{cc} k_{t}^{*}= & k_{r}\cdot k_{\left(p+16\right)}⊕k_{\left(p+16\right)}\cdot k_{\left(q+48\right)}⊕k_{r}\cdot k_{\left(q+48\right)}⊕k_{r}⊕k_{\left(p+16\right)}⊕k_{\left(q+48\right)} \end{array}$$

### 2.2. **g** Function

The variables of **g** function are $k_{t}^{\prime }$ and 16 bits of the NFSR. The feedback function of the NFSR is as follows.

(3) $$\begin{array}{cc} n_{\left(t+37\right)}= & \;k_{t}^{\prime }⊕l_{t}⊕n_{t}⊕n_{\left(t+10\right)}⊕n_{\left(t+20\right)}⊕n_{\left(t+12\right)}\cdot n_{\left(t+3\right)}\\ & ⊕n_{\left(t+14\right)}\cdot n_{\left(t+25\right)}⊕n_{\left(t+5\right)}\cdot n_{\left(t+23\right)}\cdot n_{\left(t+31\right)}\\ & ⊕n_{\left(t+8\right)}\cdot n_{\left(t+18\right)}⊕n_{\left(t+28\right)}\cdot n_{\left(t+30\right)}\cdot n_{\left(t+32\right)}\cdot n_{\left(t+34\right)} \end{array}$$

### 2.3. **f** Function

The feedback function of the LFSR is as follows.

(4) $$l_{\left(t+43\right)}=l_{t}⊕l_{\left(t+8\right)}⊕l_{\left(t+18\right)}⊕l_{\left(t+23\right)}⊕l_{\left(t+28\right)}⊕l_{\left(t+37\right)}$$

### 2.4. **h** Function

This function produces a pre-output stream from the LFSR and NFSR states as follows.

(5) $$\begin{array}{cc} h_{t}= & \;k_{t}^{*}\cdot \left(n_{\left(t+36\right)}⊕l_{\left(t+19\right)}\right)⊕l_{\left(t+6\right)}\cdot l_{\left(t+15\right)}⊕l_{\left(t+1\right)}\cdot l_{\left(t+22\right)}\\ & ⊕n_{\left(t+35\right)}\cdot l_{\left(t+27\right)}⊕n_{\left(t+1\right)}\cdot n_{\left(t+24\right)}⊕n_{\left(t+1\right)}\cdot n_{\left(t+33\right)}\cdot l_{\left(t+42\right)} \end{array}$$

### 2.5. Output Function

The output stream is produced by 5 bits of the NFSR, 1 bit of the LFSR, and the output of h function as follows.

(6) $$\begin{array}{c} z_{t}=h_{t}⊕n_{t}⊕n_{\left(t+7\right)}⊕n_{\left(t+19\right)}⊕n_{\left(t+29\right)}⊕n_{\left(t+36\right)}⊕l_{\left(t+38\right)} \end{array}$$

### 2.6. Initialization of the Cipher

The IV bits are extended to the 80 bits by concatenating 10 bits to the first of them. 1 bit one and 9 bit zeros are concatenated to the first of IV as follows.

(7) $$IV^{\prime }=1000000000v_{0}v_{1}v_{2}\ldots v_{67}v_{68}v_{69}$$

In the initialization procedure, key bits are loaded into the NFSR and LFSR from LSB to MSB ($k_{0}$ to $n_{0}$, $k_{1}$ to $n_{1}$, …, $k_{36}$ to $n_{36}$, $k_{37}$ to $l_{0}$, $k_{38}$ to $l_{1}$, …, $k_{79}$ to $l_{42}$). $c_{0}^{0}c_{0}^{1}\ldots c_{0}^{5}c_{0}^{6}$ are set to 0 in the first step of the initialization. The cipher is clocked 80 times, but before each clock, the XOR of the output bits and $IV^{\prime }$ bits are fed to the NFSR and LFSR (i.e., $z_{i}⊕v_{i}^{\prime }$, 0≤i≤79 (as show in Figure 1).

Then Cr is overwritten in the second step of the initialization, i.e., all bits of Cr are set to LSB of the NFSR except the last bit that is equal to LSB of the LFSR ($c_{80}^{0}=n_{80}$, $c_{80}^{1}=n_{81}$, …, $c_{80}^{4}=n_{84}$, $c_{80}^{5}=n_{85}$, $c_{80}^{6}=l_{80}$). Then, $l_{80}$ is set to 1 (for preventing that LFSR becomes all zeros after initialization).

In the third step of the initialization, the cipher should be clocked 80 times without the feedback in the LFSR and NFSR (i.e., during the last 80 clocks the feedback of $z_{i}⊕v_{i}^{\prime }$ is disconnected from the LFSR and NFSR). The cipher does not produce any keystream in the 160 initial clocks, i.e., $z_{0}$ to $z_{159}$ are discarded. Now the cipher is ready to produce the first bit of the keystream, i.e., $z_{160}$.

## 3. The Design Criteria

### 3.1. Limitation for Producing Keystream

Because the period of the NFSR is a multiple of $2^{43}-1$ (the period of the LFSR), the maximum length of the produced keystream is $2^{43}$ bits in each initialization. One terabyte keystream is sufficient for most applications, especially for hardware applications with the limited resource (e.g., WSN and RFID) [28].

### 3.2. Round Key Function

The key bits should participate in the internal states updating by considering two important criteria. First, the round key function should be lightweight in hardware. Second, it should be able to provide the appropriate participation of all bits of the key (as a part of the internal state) in the internal states updating. Round key function produces $2^{7}$ different keys by involving 3 bits of the key. The 3 bits are updated in every clock uniformly. If an attacker can (with guessing the internal state and known keystreams) obtain some bits of round key function, it is not easy to obtain the key bits due to the unknown counter. Round key function in Fruit-80 involves bits of the key in **g** function independently, while in Sprout cipher, none of the key bits are involved in **g** function in some clocks. Most weaknesses of Sprout were related to unsuitable round key function.

Note that we overwrite the 6 bits of Cr from NFSR values and we clock 80 times after that. If the values of Cr have been copied from LFSR, there would have been a weakness (the LFSR works independently from other sections of the cipher in the second step of the initialization).

### 3.3. **g** Function

The function that produces $n_{t+37}$ has been chosen in only 16 variables of the NFSR with regard to the light implementation in the hardware (in comparison to Grain-v1 and Plantlet). If we suppose $k_{t}^{\prime }=0$, the nonlinearity of **g** function will be $2^{3}\times 3760$ and resiliency 2. The variables for the highest degree term have been chosen from $n_{t}$ with t>27 that the degree of variables reaches the maximum possible degree in NFSR very soon.

### 3.4. **f** Function

The period of the LFSR is maximum with non-zero initial state because the feedback polynomial is primitive. Since $l_{80}$ is set to 1 after disconnecting the feedback to the LFSR (at the end of the first step of initialization), we are sure that the period of the LFSR and NFSR is at least $2^{43}-1$. Some attacks were proposed to the Grain family and Sprout from this weakness (i.e., it is possible that the LFSR becomes all zeros just before producing the first bit of the keystream in the Grain family and Sprout) [16,29].

### 3.5. Number of Clocks in the Initialization

It is very important to generate the monomial with maximal degree based on the key and IV variables in the initialization procedure, and to distribute them between all bits of the LFSR and NFSR. The numbers of clocks in the initialization procedure of Grain-v1 and Grain-128a are 160 and 256, respectively, and also the length of the NFSRs (or LFSRs) are 80 and 128, respectively. Our evaluation based on Cube attacks showed that 160 clocks is suitable for Fruit-80. It seems that 310 initial clocks is unreasonable for a lightweight cipher such as Plantlet.

### 3.6. Output Function

The nonlinearity of h function is 976 if we suppose $k_{t}^{*}=0$. We add 6 linear terms in order to increase the nonlinearity to $2^{6}\times 976=62,464$, and also to make a function with 5 resiliency. The best linear approximation of the output function has 6 terms with $2^{\left(-5.415\right)}$ bias. Note that key bits are involved in producing every bit of the keystream directly. In Plantlet cipher, it is possible to have a few clocks forward and backward with unknown key. The idea of involving directly key bits in the output increases the security margin against some attacks such as guess and determine attacks.

## 4. The Resistance to Known Attacks

The main structure of Fruit cipher was resistant to key recovery attacks since informal introduction on the web page of IACR (eprint) [23]. Two trivial key recovery attacks were proposed on Fruit. The computational complexities of the first and second attacks were $2^{82.6}$ and $2^{67.5}$ clocks of Fruit, respectively [22,24]. Fortunately, the attacks were related to the unsuitable choice of some parameters and the main structure was resistant to key recovery attacks. The design of Fruit was updated, and the attacks no longer worked. At the present time, the bottleneck of designing a secure ultra-lightweight stream cipher is TMDTO distinguishing attacks. A TMDTO distinguishing attack was successfully applied to all of the ultra-lightweight stream ciphers [24], but we propose two countermeasures that can strengthen Fruit-80 against TMDTO distinguishing attacks. A countermeasure was proposed in [24], and another countermeasure is suggested here.

### 4.1. TMDTO Attacks

It is well known that the cipher is vulnerable to these attacks if the size of its internal state is not at least twice that of the security level. This means that the number of the possible internal states (i.e., space size) should be at least $2^{160}$ for Fruit-80 after initialization (or after so many clocks) to resist against classical TMDTO attacks. The key, LFSR, NFSR, and counter are used as an internal state in Fruit-80. It is obvious that the period of a LFSR is maximum in a non-zero state. As $l_{80}$ is set to one and after that, it works independently; we are sure that the space size of LFSR is $2^{43}-1$. The space size of Cr is $2^{7}$ because it is a counter. The space size of the key is $2^{80}$ because it can take any value. The period of the NFSR is multiple of the period of the LFSR in the structure similar to Fruit-80 [30]. Thus, the space size of the NFSR is $2^{37}$. Therefore, the effective internal states are 167 bits and it is suitable.

Another condition for a stream cipher to be resistant to TMDTO attacks is good sampling resistance [31]. It means that an attacker cannot easily identify the internal states for producing the keystreams with special pattern (e.g., the keystreams that start with 10 zeros). An attacker should fix at least five variables of h function to linearize the output function and to easily identify the special internal states (it is at least two variables for Grain-v1). Therefore, it is suitable.

In the design of an ultra-lightweight stream cipher similar to Sprout and Fruit-80, it is very important to know how to benefit a key as an internal state in the internal state updating (after initialization procedure). It is obvious that all bits of the internal states (including the key for Sprout and Fruit-80) should be involved in the other internal states in a suitable ratio. The internal states excluding the key bits, participate in the internal state updating proportionally and continually in Sprout. Because the key does not affect independently (and proportionally) the internal state of Sprout, TMDTO attacks were applied successfully to Sprout [17,21]. In [17], the attacker supposes that for some consecutive clocks, the key is not involved in the internal state updating. He saves correct guesses for the internal states according to the assumption and keystreams. In the online phase of the attack, he tries to retrieve the internal states and find the key according to the keystreams. If the attacker has access to the keystreams that satisfy the assumption, he can refer to the storage and obtain the internal state and key [17]. The TMDTO attacks to the Sprout are not directly related to the main design idea of Sprout (i.e., using a key not only in initialization but also in keystream generation). It is related to the unsuitable round key function. In Fruit-80, key bits are independently involved in keystream generation and in **g** function, therefore there is no problem from this point of view. This shows that the main design idea of Sprout is not potentially weak to classical TMDTO attacks, and also, Fruit-80 can be resistant to classical TMDTO attacks.

#### TMDTO Distinguishing Attacks

If it is supposed that key is fixed in Grain-v1, the internal state space is equal to twice of the security level (i.e., $2^{160}$). For a small-state stream cipher, the internal state space is less than twice of the security level under a fixed key. This opens a door to TMDTO distinguishing attacks to small-state stream ciphers. Without any countermeasure for the TMDTO distinguishing attacks on Fruit-80, an attacker can distinguish keystreams of Fruit-80 from truly random sequences (under every arbitrarily fixed key) with data, time and memory complexity of $2^{50.2}$ bits of keystream, $2^{52.4}$ clocks of the cipher and $2^{50.2}$ bits memory, respectively [24]. As discussed in the introduction, we ruled out TMDTO distinguishing attacks against Fruit-80 (similar to Fruit and Plantlet ciphers [23,26]). Nevertheless, two countermeasures are proposed to strengthen Fruit-80 against all types of TMDTO attacks as follows.

**Permanently IV mixing**. In [24] a new idea was proposed to strengthen the small-state stream ciphers against TMDTO distinguishing attacks. The new idea was to use IV bits not only in the initialization but also in the keystream generation. In other words, the new idea was permanently using IV (similar to the key) in the internal state updating [24]. It is obvious that key should be saved for reuse by other IVs in the most real world stream ciphers. Thus, the use of the key does not impose any area in hardware (except for accessing the key bits). Furthermore, permanently using IV does not impose any area in some applications. One famous example is A5/1 stream cipher in the GSM system. Allowed IVs, which are 22-bit frame numbers, only one time are produced for every key (reusing the same IV is strictly prohibited with the same key in all stream ciphers). A 22-bit memory is dedicated to storing the IV (i.e., frame numbers) that it increases in every loading sequentially. On the other hand, it is a general structure that parameter(s) (e.g., packet number) is stored in a memory as IV in cryptosystems. In this structure, there is no vulnerability regarded from using the same IVs twice with the same key [24].

**Limitation on the number of keystream bits per key.** If less than $2^{17}$ keystream bits are produced under every key, then TMDTO distinguishing attacks are not applicable on Fruit-80 with the time and data complexities significantly smaller than that of brute force attack. Suppose that we produce $2^{16}$ keystream bits under every key. In the first step of the attack, an attacker saves $2^{15}$ keystream bits of Fruit-80 under a fixed key in a searchable table. In the second step, the attacker produces next $2^{15}$ keystream bits of Fruit-80 under the fixed key, and he searches for a collision in the table. The probability that he cannot find a collision is as follows.

(8) $${\left(1-\frac{2^{15}}{2^{87}}\right)}^{2^{15}}$$

Note that there are $2^{87}$ different internal states under a fixed key. The attacker can repeat the first and second steps for $2^{57}$ different keys. In this case, he will be able to find a collision with probability of about 0.63 as follows.

(9) $$1-{\left({\left(1-\frac{2^{15}}{2^{87}}\right)}^{2^{15}}\right)}^{2^{57}}=1-{\left(1-\frac{1}{2^{72}}\right)}^{2^{72}}\approx 0.63$$

The data and computational complexity of this attack are $2^{16}\times 2^{57}\times 87=2^{79.4}$ and $2^{16}\times 2^{57}=2^{73}$, respectively. The number of IVs is not important in the limitation. It means that $2^{16}$ keystream bits can be produced under one or many IVs. As many as the number of produced keystream bits is increased, the security decreases against TMDTO distinguishing attacks. Thus, Fruit-80 will be resistant against TMDTO distinguishing attacks if up to $2^{16}$ keystream bits are produced under every key. Note that some applications do not require so many keystream bits in its lifetime, and in some other applications, it is possible to reinitialize ciphers after producing limited keystream bits with a new key. In both of them, it is practical to accept this limitation because small-state stream ciphers are for constrained environments. For example, it usually needs few keystream bits in the lifetime of a WSN or RFID. In Lizard cipher, designers are allowed to produce up to $2^{18}$ keystream bits per key/IV pair, and they mentioned that $2^{18}$ keystream bits are sufficient for many existing scenarios like Bluetooth, WLAN, and HTTPS [24]. Thus, this countermeasure can strengthen every small-state stream cipher against TMDTO distinguishing attacks. We discuss in the following that Fruit-80 is secure against all types of key recovery attacks without any limitation on the number of keystream bits.

### 4.2. Guess and Determine Attacks

Due to the small internal state in Fruit-80 and the weakness of Sprout against these attacks [16], these attacks are important. If an attacker guesses all bits of the internal state in Sprout, he can clock two times forward and one time backward (with unknown key), and he can decrease the wrong candidates of the internal state in each clock. In the next clocks, the attacker obtains one bit of the key or decreases the wrong candidates. In Fruit-80, key bits are involved in the output function to prevent producing the keystream in the next and previous clock with unknown key.

If an attacker wants to produce only the first 8 bits of the keystream in Fruit-80, he needs to guess all bits of the LFSR and NFSR. We suppose that he guesses all bits of the LFSR and NFSR. He can obtain one bit of $k_{t}^{*}$ in the next clock. There are two unknown variables (i.e., $k_{t}^{\prime }$ and $k_{t}^{*}$) in the next clocks. It is too hard for him to solve equations and obtain key bits with regard to the unknown Cr. There are 256 different $k_{t}^{\prime }$ and $k_{t}^{*}$ bits. Thus, the attacker should guess the bits of Cr. Note that the 7 bits of the NFSR and LFSR are copied to Cr during the initialization and after that, the cipher is clocked 80 times. It means that many times the value of Cr are changed based on the key, LFSR, and NFSR. Therefore, it is impossible for the attacker to obtain valuable information about Cr from the guessed values.

If an attacker guesses all bits of the LFSR, NFSR, and Cr i.e., 87 bits, he can obtain the round key function bits in Fruit-80. The attacker can obtain two equations in each clock and provide a nonlinear equation system involving unknown key bits. He needs to clock at least 80 times the cipher. It is impossible for the attacker to identify wrong candidates of the internal state before 80 clocks. In this situation, the attacker should solve the nonlinear equation system, or he should try to identify wrong candidates (note that both of them are not easy). Thus, the complexity of this attack is higher than that of exhaustive search attack. Thus, Fruit-80 is resistant to this attack.

We analyzed how an attacker can use the copied values in other scenarios in the second step of initialization. If the attacker guesses all bits of the LFSR and NFSR at the end of the first step of the initialization, he also has the values of Cr. If he considers the round key function bits as some unknown variables, he can obtain an equation system. In this scenario, the attacker guesses the 80 bits and he should clock 160 times (80 clocks for the third step of the initialization and 80 clocks for applying all bits of the key). The degree of equations increases very fast, and the computational complexity of this attack is more than that of exhaustive search attack.

### 4.3. Linear Approximation Attacks

A linear approximation attack was applied to Grain-v0 [32]. In [32] it has been discussed that if the NFSR and the output function are chosen with high nonlinearity and suitable resiliency, the ciphers similar to Grain-v0 will be resistant to linear approximations attacks. We choose the NFSR and output function with high nonlinearity and good resiliency, and also a nonlinear function of the key is involved on the NFSR and output function. If we suppose $k_{t}^{*}=0$, the best linear approximation of the output has $2^{\left(-5.415\right)}$ bias as follows.

(10) $$z_{t}=n_{t}⊕n_{\left(t+7\right)}⊕n_{\left(t+19\right)}⊕n_{\left(t+29\right)}⊕n_{\left(t+36\right)}⊕l_{\left(t+38\right)}$$

If we suppose $k_{t}^{\prime }=0$, the best linear approximation of the NFSR feedback function has $2^{\left(-4.6\right)}$ bias as follows.

(11) $$n_{\left(t+37\right)}=n_{t}⊕n_{\left(t+10\right)}⊕n_{\left(t+20\right)}⊕l_{t}$$

If an attacker eliminates the NFSR bits between these two relations (by shifting and XORing of the linear approximation of the output), he can obtain the following relation with $2^{\left(-36.66\right)}$ bias (by Piling-up Lemma).

(12) $$\begin{array}{cc} z_{t}⊕z_{\left(t+10\right)}⊕z_{\left(t+20\right)}⊕z_{\left(t+37\right)}= & \;l_{t}⊕l_{\left(t+7\right)}⊕l_{\left(t+19\right)}⊕l_{\left(t+29\right)}⊕l_{\left(t+36\right)}\\ & ⊕l_{\left(t+38\right)}⊕l_{\left(t+48\right)}⊕l_{\left(t+58\right)}⊕l_{\left(t+75\right)} \end{array}$$

Now, if the attacker tries to obtain a relation only based on the keystream bits (by using the feedback function of LFSR), the bias will be too small. Therefore, Fruit-80 is resistant to linear approximation attacks.

### 4.4. Related-Key Attacks

There are weaknesses in the initialization procedure of all members of the Grain family [4,14] and Sprout [18,33]. Designers of Plantlet ruled out the related-key attacks. They believed related-key attacks are not workable on Plantlet because the key is fixed in ultra-lightweight ciphers [26]. Nevertheless, we propose a new scheme in the initialization procedure to strengthen Fruit-80 against these attacks. We did not load the IV bits directly in the internal state and did not combine the IV and key bits straightforward together (e.g., First bit of the IV (i.e., $v_{0}$) is XORed with the 11th bit of the output). We use key bits in the round key function and also we load in the LFSR and NFSR. Thus, the new idea increases the resistance to the related-key attacks. In Grain-v1, key bits are loaded into the NFSR, and IV bits are loaded into the LFSR. Key and IV bits use only one time and combine together directly in Grain-v1 [3]. Thus, Fruit-80 is resistant to related-key attacks by exploiting the new idea and asymmetric padding in the IV bits loading.

### 4.5. Cube Attacks

A type of Cube attack (i.e., dynamic Cube attack) was applied to Grain-128 [6,8] because the degree of NFSR feedback was low, i.e., 2. The degree of NFSR feedback was increased to 4 in Grain-128a to compensate this weakness [12]. According to the suitable clock number of Fruit-80 in the initialization procedure and the degree of NFSR feedback, it is impossible to find low degree multiplicative expressions (of some bits of the IV) based on the key in the Boolean function of the output. In Fruit-80, the length of LFSR and NFSR are shorter than that of Grain-v1, and also there are 13 variables in the h function of Fruit-80 while there are five variables in the h function of Grain-v1. Thus, it is acceptable that the degree of key and IV variables in the initialization procedure of Fruit-80 grows faster than that of Grain-v1. As the number of clocks in the initialization of Fruit-80 is equal to that of Grain-v1 (and the length of the FSRs are shorter than that of Grain-v1), Fruit-80 is likely stronger than Grain-v1 against Cube attacks. Nevertheless, we implemented a Cube attack with Cube size of 32 bits (i.e., we XOR the first bits of the keystreams for all possible values of 32 bits of the IV, and we suppose that other bits of IV are zeros) for 100 and 80 initial clocks. The superpolys were not linear or constant over the key variables. The results show that Fruit-80 (with 160 initial clocks) is resistant to all types of Cube attacks.

### 4.6. Algebraic Attacks

Algebraic attacks have not been applied to the Grain family, but a type of algebraic attack was applied to Sprout [20]. Unsuitable round key function in Sprout has caused this weakness. The key is not involved in the internal state updating (and consequently in the keystream generation) in half of clocks. An attacker can guess some bits of the NFSR and LFSR and obtain equations for the keystream generation involving the unknown round key function bits. Then, he can solve the equation system and obtain the key bits. In the clocks in which the key is not involved in the internal state updating, he has the chance to identify a bit of LFSR in the next clock of Sprout. Due to the direct impact of the key on the round key function of Fruit-80, this attack is not workable on Fruit-80. On the other hand, the attacker should guess almost all bits of the LFSR and NFSR and Cr to obtain an equation system based on the key bits.

It is impossible for an attacker to apply pure algebraic attacks because the degree of polynomials in the internal state of Fruit-80 grows very fast. Here we discuss that a combination of guess and determine attacks with algebraic attacks is not applicable to Fruit-80.

If an attacker guesses bits of NFSR, Cr and round key function, then he can obtain two equations in each clock (one from the keystream generation and one from the round key function). These equations are degree 2 and 3. It is not easy to solve the equations, but we suppose that the attacker can solve equations of keystream and obtain 1 bit of LFSR in each clock. In this scenario, he should guess at least 42 bits of the round key function. Then, he can obtain the next bits of the round key function. Totally, the attacker should guess 37 + 7 + 42 bits and he should clock (at least) 80 times for each guess and should solve two non-linear equation system (one for LFSR and one for key). Thus, the computational complexity of the attack is more than that of exhaustive search attack.

### 4.7. Fault Attacks

These attacks have been applied successfully to all members of Grain family [13,34] and also to Sprout [20,33]. Fault attacks are based on some impractical hypotheses. An attacker should be able to induce a fault in the cipher in a special time and supposes that the induced fault affects only a special section of the cipher, e.g., the attacker should be able to induce a single bit fault in the NFSR just after initialization [33]. Another unrealistic hypothesis is that the attacker can reset the cipher and obtain the correct keystream (i.e., keystream with same key and IV, but without inducing the fault).

It is possible to protect both LFSR and NFSR in the hardware implementation from inducing fault by using a mirror or mask in the hardware [35].

### 4.8. Weak Key-IVs

There are weak key-IVs in all members of the Grain family [29] and Sprout [16,33]. It is possible that all bits of the LFSR become zeros before producing the first bit of the keystream. In this situation, the LFSR remains at all zeros for all clocks, and NFSR statistical properties will become non-random. As a result, the period of the cipher is unknown, and the keystream is only dependent on NFSR bits, and the cipher is much too vulnerable. This is very important in small-state ciphers with regard to shorter LFSRs. Fortunately, because we set $l_{80}$ to 1 in the second step of the initialization, and after that the LFSR works independently, it is impossible that all bits of the LFSR become zeros in Fruit-80. Thus, there is no weak key-IV in Fruit-80.

## 5. Hardware Implementation

The design of lightweight cipher is very important in industry, while we need light ciphers in various fields such as WSN, WBAN, and RFID. Thus, our goal was to design a strong cipher with less than 80% GE of the hardware implementation of Grain-v1 (in the same condition) as an ultra-lightweight stream cipher. To get the area size of Grain-v1, Plantlet, Lizard, and Fruit-80 in the hardware, we simulated them by Modelsim SE 10.2c, and we used TSMC 0.18 μm technology and the Synopsys Design Compiler 2013.03 sp2 version for synthesis and optimization. As Sprout was insecure and Plantlet is the improved version of it, we did not implement Sprout.

In Table 1, we compare the area size of the hardware implementation of Fruit-80 and some other lightweight stream ciphers. The area size of Fruit-80 is significantly less than Grain-v1, as expected with regard to the size of the internal state (the internal state of Grain-v1 is 160 bits, but for Fruit-80 is 87 bits). Note that one GE is equivalent to the area of a 2-way NAND gate. Table 1 shows that the area size of Grain-v1 is about 32% bigger than that of Fruit-80 in our results.

We did not dedicate any GE for storing the key bits (such as implementation of Sprout and Plantlet in [15,26]) because key (for reuse with different IVs) should be stored in most applications and it is possible to store the fixed key bits in a non-volatile memory, e.g., burn the key in a fuse (that needs less area size in comparison to storing bits in a volatile memory [15]).

Note that IV bits are loaded into LFSR in Grain-v1 at once; but in Fruit-80, IV bits are XORed bit by bit with the output in the initialization step. This new idea for loading IV bits has some advantages. All bits of IV should be in access to start loading in Grain-v1 (and in many stream ciphers such as Plantlet [26], Trivium [1], and WG-8 [37]), but in Fruit-80 (and in some stream ciphers such as A5/1 [38] and MICKEY 2.0 [2]) initialization can be started as soon as the first bit of the IV is available. Thus, the speed of the initialization of Fruit-80 is higher than that of Grain-v1 while IV is not available at once. This is important because IV bits are produced one by one in some cases. We did not consider any memory for storing the IV bits in the case that all bits of IV are available at once. In this case, we suppose that there is a memory for storing IV bits in the where that IV bits are produced in out of Fruit-80.

Another advantage of Fruit-80’s IV loading is that it is more resistant to related-key attacks than that of Grain-v1 and Plantlet (as already discussed).

## 6. Conclusions

The storing of key bits for reuse by different IVs is essential for most applications, and also it is necessary to store a key in a fixed memory in some applications (e.g., in RFID systems or SIM cards for mobile phones). It is a valuable idea that the stored key is also used in the design as a part of the internal state. This idea helped us to design an ultra-lightweight stream cipher with a significantly smaller area size (i.e., Fruit-80).

We presented the hardware implementation results and compared the area sizes of some stream ciphers. Fruit-80, Plantlet, Lizard, and Grain-v1 require 960, 996, 1218, and 1270 GE for implementations in TSMC 0.18 μm technology, respectively. These results are expected because the size of the internal states of Fruit-80, Plantlet, Lizard, and Grain-v1 are 87, 101, 121, and 160 bits, respectively. The results show that Fruit-80 is the lightest small-state stream cipher and the area size of Grain-v1 is about 32% bigger than that of Fruit-80. As Grain-v1 is the lightest candidate in the hardware profile of eSTREAM project, it is obvious that design of secure stream ciphers as small as Fruit-80 is attractive.

A TMDTO distinguishing attack was successfully applied to all of the small-state stream ciphers. A new countermeasure to strengthen small-state stream ciphers against TMDTO distinguishing attacks was proposed in this work. The new idea is producing up to $2^{16}$ keystream bits under every key, which is practical in most cases, because small-state stream ciphers are mostly for constrained environments.

## Figures and Tables

**Table 1:** The area size of Fruit-80 and some lightweight stream ciphers.

Cipher	Area Size (GE)	Throughput ${\left(\mathrm{Kb}/s\right)}^{\;\Omega }$	Platform	Source
Mickey [2]	3188	100	0.13 μm CMOS	[36]
Trivium [1]	2580	100	0.13 μm CMOS	[36]
Grain-v1 [3]	1294	100	0.13 μm CMOS	[36]
Lizard [27]	1161	100	0.18 μm CMOS	[27]
Grain-v1 [3]	1221	100	0.18 μm CMOS	[36]
Plantlet [26]	928	100	0.18 μm CMOS	[26]
Grain-v1 [3]	1162	100	0.18 μm CMOS	[26]
Grain-v1 [3]	1270	100	0.18 μm CMOS	Our work
Lizard [27]	1218	100	0.18 μm CMOS	Our work
Plantlet [26]	996	100	0.18 μm CMOS	Our work
Fruit-80	960	100	0.18 μm CMOS	Our work

^Ω^ The throughput is for the clock of 100 KHz frequency.

## Appendix A. Test Vector for Fruit-80

We use hexadecimal format for presenting the keystream, key, and IV as follows.

$K=\{k_{0}k_{1}k_{2}k_{3},\;k_{4}k_{5}k_{6}k_{7},\;k_{8}k_{9}k_{10}k_{11},\ldots ,\;k_{72}k_{73}k_{74}k_{75},\;k_{76}k_{77}k_{78}k_{79}\}$

$IV=\{00v_{0}v_{1},\;v_{2}v_{3}v_{4}v_{5},\;v_{6}v_{7}v_{8}v_{9},\ldots ,\;v_{62}v_{63}v_{64}v_{65},\;v_{66}v_{67}v_{68}v_{69}\}$

$Z=\{z_{160}z_{161}z_{162}z_{163},\;z_{164}z_{165}z_{166}z_{167},\;z_{168}z_{169}z_{170}z_{171},\ldots \}$

First test vector:

k={0,0,0,0,0,0,0,0,0,0,0,0,0,0,0,0,0,0,0,0}

IV={0,0,0,0,0,0,0,0,0,0,0,0,0,0,0,0,0,0}

Z={9,D,6,3,4,B,D}

Second test vector:

k={0,0,0,0,0,0,0,0,0,0,0,0,0,0,0,0,0,0,0,1}

IV={0,0,0,0,0,0,0,0,0,0,0,0,0,0,0,0,0,1}

Z={5,E,C,5,1,0,D}
